# Mediation analysis of erythrocyte lipophilic index on the association between BMI and risk of oral cancer

**DOI:** 10.1186/s12944-022-01704-z

**Published:** 2022-10-08

**Authors:** Yi Fan, Qing Chen, Yaping Wang, Jing Wang, Yanni Li, Sijie Wang, Yanfeng Weng, Qiujiao Yang, Chen Chen, Lisong Lin, Yu Qiu, Fa Chen, Jing Wang, Baochang He, Fengqiong Liu

**Affiliations:** 1 Department of Epidemiology and Health Statistics, Fujian Provincial Key Laboratory of Environment Factors and Cancer, School of Public Health, Fujian Medical University, 1 Xueyuan Road, Fuzhou, 350122 China; 2 Key Laboratory of Ministry of Education for Gastrointestinal Cancer, Fujian Key Laboratory of Tumor Microbiology, Fujian Medical University, Fujian, China; 3 School of Public Health, Zunyi Medical University, Zunyi, Guizhou China; 4 Department of Oral and Maxillofacial Surgery, the First Affiliated Hospital of Fujian Medical University, Fujian, China; 5 Laboratory Center, The Major Subject of Environment and Health of Fujian Key Universities, School of Public Health, Fujian Medical University, Fujian, China

**Keywords:** Fatty acids, Membrane fluidity, Lipophilic index, Oral cancer, Mediation analysis

## Abstract

**Aims:**

To explore the relationship between the fatty acid lipophilic index (LI) of the erythrocyte membrane and oral cancer risk, as well as to evaluate the possibility of LI acting as a mediator of the association between body mass index (BMI) and oral cancer.

**Method:**

Twenty-three fatty acids (FAs) of the erythrocyte membrane were measured using gas chromatography in 380 patients with oral cancer and 387 control subjects. The LI was calculated based on the FA proportion and FA melting points. The association of BMI and erythrocyte LI with oral cancer risk was analysed using logistic regression. The mediation effect of LI on the association between BMI and oral cancer risk was evaluated using mediation analysis.

**Results:**

Among the control group, 46.0% were overweight or obese, which was significantly higher than that of oral cancer patients (29.5%). Significant differences in erythrocyte membrane saturated fatty acids (SFAs), monounsaturated fatty acids (MUFAs), and polyunsaturated fatty acids (PUFAs) were observed between the patient and control groups. The proportion of C18:1 n-9 from the MUFA family increased in oral cancer patients (12.67%) compared with controls (12.21%). While the total proportion of n-3 PUFAs decreased in oral cancer patients compared with controls, with C20:5 n-3 decreasing from 0.66 to 0.47%, and C22:6 n-3 decreasing from 5.82 to 4.86%. The LI was lower in the control participants (M = 27.6, IQR: 27.3–27.9) than in the oral cancer patients (M = 28.2, IQR: 27.9–28.5). BMI was inversely associated with oral cancer risk with a fully adjusted *OR* of 0.59 (95% *CI*: 0.43–0.83), while LI was positively associated with oral cancer risk with a fully adjusted *OR* of 1.99 (95% *CI*:1.36–2.94). LI explained 7% of the variance in the relationship between BMI and oral cancer risk.

**Conclusions:**

The distribution of the FA profile in erythrocyte membranes differed between the oral cancer patients and the control group. The LI derived from the profile of FAs was positively associated with the risk of oral cancer, and the associations between BMI and oral cancer risk can be explained, at least in part, by LI.

**Supplementary Information:**

The online version contains supplementary material available at 10.1186/s12944-022-01704-z.

## Introduction

Oral cancers, are a common cancer type globally, and approximately 377,713 new cases occur every year [1]. In China, oral cancer was frequently overlooked by governments in public health policy, although it had emerged as a substantial component of worldwide oral health issues, with considerable societal and economic consequences [2]. In addition to smoking, drinking, poor oral hygiene, and human papillomavirus, which have all been identified as causative factors in oral cancer [3–5], studies have also reported that BMI plays a crucial role in oral cancer [6–8]. Changes in BMI have been shown to affect metabolic lipid profiles and studies have indicated that obesity is a product of metabolic imbalance, as well as a driver of fatty acid (FA) metabolism disturbance [9]. Elevated levels of free FA and dyslipidemia are common in obesity. Obesity-related genes and lipid signaling are dysregulated in the microenvironment, exacerbating lipid metabolism problems and facilitating tumor proliferation and development in obese patients [10, 11].

FA composition in the erythrocyte membrane is regarded as a comprehensive biomarker of the homeostatic condition of FA. FA composition in membranes determines the fluidity of the membranes, which subsequently impacts the capacities of membrane-bound proteins and cells [12]. Moreover, the FA profile of the erythrocyte membrane is representative of the general condition of other tissues which coordinates the complex interaction between dietary intake of FAs and endogenous FA metabolism [13]. In several studies, abnormalities in FA profiles have been reported to be linked to chronic diseases including cancers [14–16]. Furthermore, a few studies have looked at the impact of changing FA levels on the etiology of malignancies, using the erythrocyte membrane as a possible biomarker [16–20]. The lipophilic index (LI), which is a measurement of the lipophilic appeal of different FAs, may be determined primarily from the FA composition of biological membranes. The LI was defined as the average of the melting points of individual FAs multiplied by the corresponding proportion in the membrane. Previous studies have discovered the relationship between LI and chronic diseases such as diabetes and coronary heart disease [13, 21, 22].

In the current study, the association between BMI, erythrocyte membrane LI and oral cancer, as well as whether LI could mediate the association between BMI and oral cancer risk, were explored.

## Materials and methods

### Study population and data collection

A hospital-based case-control study was conducted with patients from the First Affiliated Hospital of Fujian Medical University from September 2013 to December 2020 in Fujian Province, China, as previously described [23]. Patients with newly diagnosed oral cancer (C00-C07 corresponding to the 10th revision of the International Classification of Diseases) were enrolled in this study and serum samples were collected at admission. The criteria for inclusion were as follows: (1) primary oral cancer diagnosed histologically; (2) Chinese population with at least 10 years of residency in Fujian Province; and (3) age between 20 and 80 years. Those with recurring or metastasized cancer, as well as those who had already received chemotherapy or radiation, were excluded. During the same period, control subjects were recruited from the physical examination center of the same hospital. Any additional malignancies that were present or had been present were excluded. Finally, 380 patients and 387 healthy participants were admitted into the study.

The study protocol was accepted by Fujian Medical University’s Ethics Committee (Approval number:2011053; Approval date: March 10, 2011) and followed the Declaration of Helsinki’s ethical standards. All participants gave their informed consent. For both the case and control groups, demographic data were obtained by face-to-face interviews using a standardized questionnaire.

### BMI assessment

Height and weight were measured by the nurse of the hospital at admission. Body mass index (BMI) was calculated as weight (in kilograms) divided by the square of the height (in meters) and was classified into three categories (< 18.5 /18.5 ~ 23.9 /≥24).

### Measurement of the FA profile of erythrocytes membranes and calculation of LI

Blood samples from the study population in a fasting state at their first admission were collected. Venous blood samples (approximately 5 mL) collected in vacutainer tubes were used to determine the FA composition of mature erythrocyte membrane phospholipids. The samples were kept at − 80 °C until they were analyzed.

Details of the erythrocyte membrane FA acquisition and measurement have been described previously [23]. Briefly, FA methyl esters were separated using an Agilent 7890B gas chromatograph (Agilent, Palo Alto, CA, USA) equipped with a DB-23 fame column (60 m × 0.25 mm inside diameter, the film thickness of 0.15 μm) for flame ionization. A mixed FA methyl ester standard (Sigma-Aldrich Inc., USA) was used for the separation of FA methyl ester peaks. A total of 38 erythrocyte membrane FAs were tested, and 23 erythrocyte membrane FAs were above the detection limit. The FA composition is presented as the percentage of individual FAs divided by the total FAs.

The LI was calculated using the following equation [21]:

$$\mathrm{Lipophilic}\ \mathrm{index}=\frac{\ \sum_k\Big[ Levels\ of\ fatty\ acid\left(\%\right)i\times melting\ point\left({}^{{}^{\circ}}C\right)i\Big]}{\sum_k\left[ Levels\ of\ fatty\ acid\left(\%\right)i\right]}$$

where *i* denotes individual FAs and *k* is the total number of FAs utilized to compute LI. This index reflects the overall FA lipophilicity in erythrocytes.

The LipidBank database (http://lipidbank.jp/) was used to obtain the melting points of FAs. Of the 23 quantified individual FAs in erythrocytes, data on melting points were available for 20. Hence, 20 FAs were included to calculate the LI (8% of measured FAs not considered, 92% of measured FAs considered).

### Covariate analysis

Two educational level groups were defined: the low group (lower vocational training or primary school) and the high group (secondary school and above). Demographic factors included age (< 60 years/≥60 years), sex (male/female), residence (rural/urban), marital status (married/others), education (low/high), occupational activity (light, moderate, or heavy) [24], family history of cancers (no/yes), smoking status (no/yes), alcohol drinking (no/yes), diabetes (no/yes) and oral hygiene (well/poor).

### Statistical analysis

The distribution of demographic characteristics between the case and control groups was examined using the χ2 test or Fisher’s exact test. The distribution of FAs between patients and control participants was analyzed using the Wilcoxon rank-sum test and visualized by a violin plot. The correlations between individual FAs and the LI were investigated using Spearman’s correlation coefficients.

The association between BMI, erythrocyte membrane LI and oral cancer was analyzed using an unconditional logistic regression model with BMI and LI being treated as both categorical and continuous variables. *OR*s and 95% *CI* were calculated. In addition to the crude model, age, sex, education, residence, marital status, occupational activity, and family history of cancer were adjusted for in Model 2. Model 3 was further adjusted for smoking status, alcohol drinking, oral hygiene, and history of diabetes. Mediation analysis was performed to test the mediation effect of LI on the association between BMI and oral cancer using the medflex package in R. Covariates in mediation analysis were obtained using a generalized linear model with the mediated model (BMI → LI) and outcome model (LI and BMI → oral cancer). Analysis of the study was performed in R version 4.1.0.

## Results

The baseline characteristics of the study population are shown in Table 1. There were 387 control subjects and 380 patients in the study. The mean BMIs of the control and oral cancer cases were 23.78 kg/m^2^ and 22.25 kg/m^2^, respectively. In the control group, the proportion of overweight or obese was 46.0%, while in the oral cancer group, the proportion of overweight or obese was 29.5%. No statistically significant differences were observed in the distribution of sex, residence, marital status, or occupational activity between the patient and control groups (all *P* > 0.05). Compared with the control group, the case group was characterized by a higher proportion of subjects with smoking, drinking, and family history of cancer.

**Table 1:** Baseline characteristic of the study participants

Characteristic	Controls (*n* = 387) n(%)	Oral cancer (*n* = 380) n(%)	*P*
Sex			0.197
Male	186 (48.1%)	165 (43.4%)	
Female	201 (51.9%)	215 (56.6%)	
Age			0.001
< 60	151 (39.0%)	192 (50.5%)	
≥60	236 (61.0%)	188 (49.5%)	
BMI			< 0.001
< 18.5	14 (3.6%)	39 (10.3%)	
18.5 ~ 23.9	195 (50.4%)	229 (60.2%)	
≥24	178 (46.0%)	112 (29.5%)	
Education			< 0.001
low	142 (36.7%)	56 (14.7%)	
high	245 (63.3%)	324 (85.3%)	
Residence			0.536
Rural	193 (49.9%)	198 (52.1%)	
Urban	194 (50.1%)	182 (47.9%)	
Marital status			0.093
Married	358 (90.4%)	381 (93.6%)	
Others	38 (9.6%)	26 (6.4%)	
Occupational activity			0.329
Light	77 (19.9%)	91 (23.9%)	
Moderate	180 (46.5%)	175 (46.1%)	
Heavy	130 (33.6%)	114 (30.0%)	
Smoking status			0.002
No	275 (71.1%)	229 (60.3%)	
Yes	112 (28.9%)	151 (39.7%)	
Alcohol drinking			< 0.001
No	315 (81.4%)	263 (69.2%)	
Yes	72 (18.6%)	117 (30.8%)	
Family history of cancer			0.026
No	340 (87.9%)	312 (82.1%)	
Yes	47 (12.1%)	68 (17.9%)	
Diabetes			< 0.001
No	295 (76.2%)	336 (88.4%)	
Yes	92 (23.8%)	44 (11.6%)	
Oral hygiene			0.020
Well	113 (29.2%)	141 (37.1%)	
Poor	274 (70.8%)	239 (62.9%)	

The melting points, proportions of each erythrocyte membrane FA, and Spearman’s correlation coefficients between the individual FAs and LI were listed in Table 2. The major FA components of the erythrocyte membrane were C16:0 (22.83%), C18:0 (12.40%), C18:1 (12.38%), C18:2 (12.48%) and C20:4 (13.31%). Generally, erythrocyte LI was positively correlated with SFAs, including C16:0, C18:0, C20:0, C22:0, C23:0, and C24:0, and MUFAs, including C18:1 n-9, C22:1 n-13, C24:1 n-15 (all r > 0, *P* < 0.05). However, PUFAs, including C20:5 n-3, C22:5 n-3, and C22:6 n-3, were inversely correlated with the LI (all r < 0, *P* < 0.05).

**Table 2:** Fatty acid melting points, erythrocyte fatty acid mean proportions, and erythrocyte FA correlation coefficients with the Lipophilic Index (LI)

FA	Melting point (°C)^a^	Mean proportion (%)^b^	Correlation with LI (Spearman)
SFAs			
C14:0	53.9	0.34	−0.030
C15:0	52.3	0.11	0.027
C16:0	63.1	22.83	0.564**
C17:0	61.3	0.32	0.048
C18:0	69.6	12.40	0.269**
C20:0	76.8	0.39	0.144**
C22:0	81.5	1.11	0.104**
C23:0	79.1	0.15	0.084*
C24:0	87.8	3.47	0.415**
MUFAs			
C16:1 n-9	0	0.40	0.017
C18:1 n-9	16	12.38	0.266**
C20:1 n-9	23.3	0.54	0.069
C22:1 n-13	34.7	0.19	0.095**
C24:1 n-15	42.8	3.82	0.564**
PUFAs			
n-6 PUFAs			
C18:2 n-6	−5	12.48	0.170**
C20:4 n-6	−49.5	13.31	−0.010
n-3 PUFAs			
C18:3 n-3	−11.2	0.06	0.030
C20:5 n-3	−54.1	0.61	−0.655**
C22:5 n-3	−54.1	2.50	−0.547**
C22:6 n-3	−44.2	4.77	−0.782**

* Correlation was significant at the 0.05 level

** Correlation was significant at the 0.01 level

^a^ Values obtained from the LipidBank database (http://lipidbank.jp/)

^b^ Relative mean concentration of fatty acids (%)

**Fig. 1:**
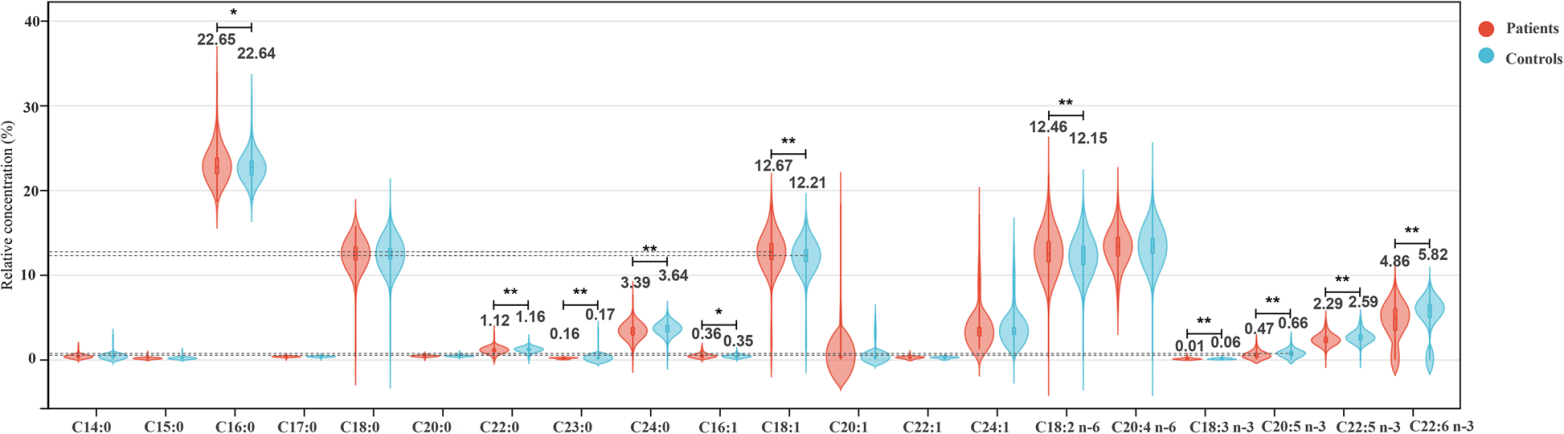
Distribution of fatty acids between patients and control participants (*Correlation was significant at the 0.05 level; ** Correlation was significant at the 0.01 level)

The FA composition of erythrocyte membranes in the patient and control groups was shown in Fig. 1. Significant differences in saturated FAs (including C16:0, C22:0, C23:0, and C24:0), monounsaturated FAs (including C16:1 n-9 and C18:1 n-9), n-6 polyunsaturated FAs (including C18:2 n-6) and all n-3 polyunsaturated FAs (including C18:3 n-3, C20:5 n-3, C22:5 n-3, and C22:6 n-3) were observed between the two groups (all *P* < 0.05). The proportion of C18:1 n-9 from the MUFA family was increased in oral cancer patients (12.67%) compared with controls (12.21%). The total proportion of n-3 PUFAs was decreased in oral cancer patients compared with controls, with C20:5 n-3 decreasing from 0.66 to 0.47% and C22:6 n-3 decreasing from 5.82 to 4.86%.

**Fig. 2:**
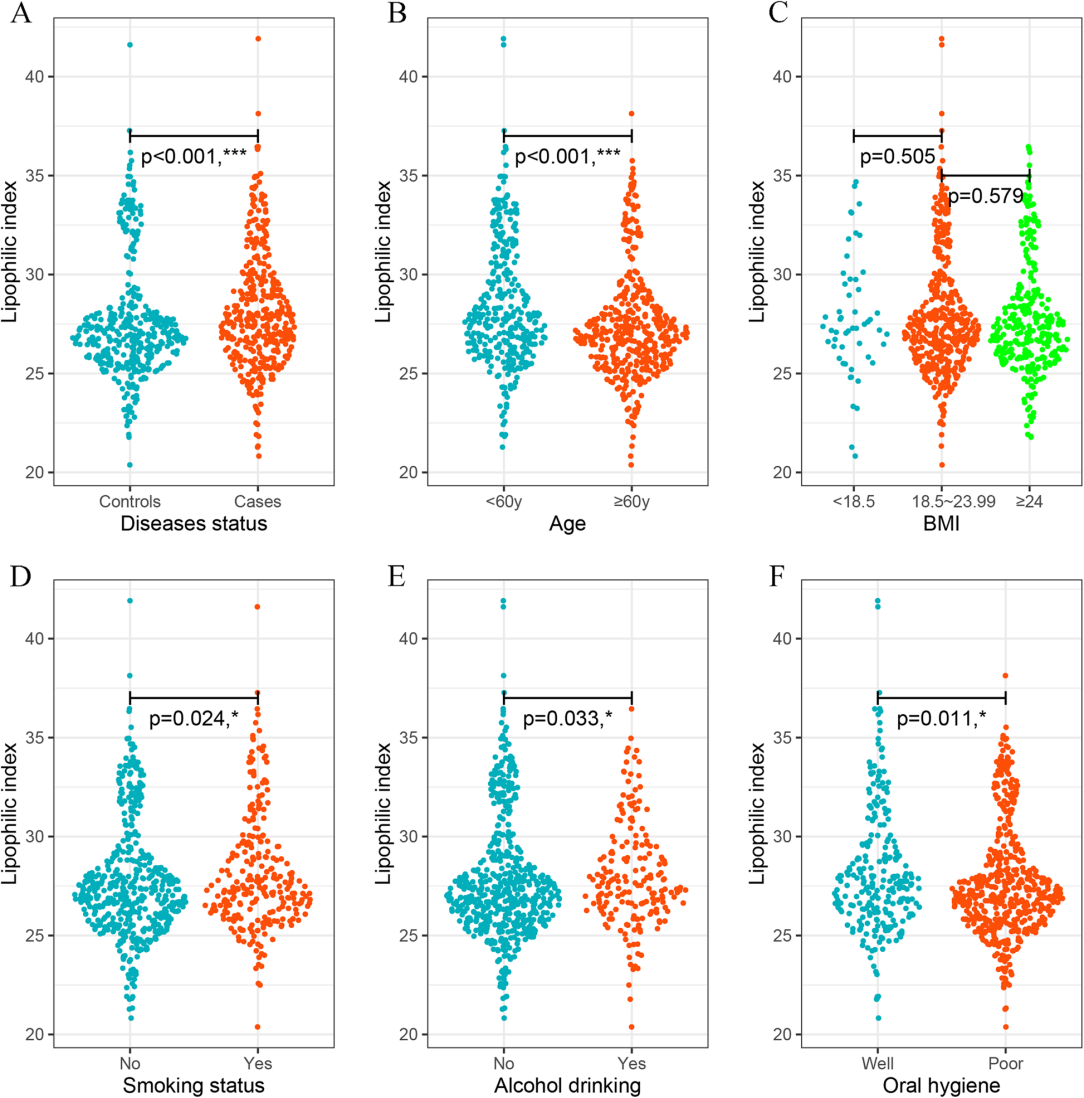
Classified scatter plots of the lipophilic index according disease status, age, BMI, smoking status, alcohol drinking, and oral hygiene (**A**) by disease status (Median(IQR): 26.9(25.8,28.1) vs. 27.7(26.2,27.7) (**B**) by age (Median(IQR): 27.7(26.3,30.3) vs. 26.9(25.7,28.2) (**C**) by BMI (Median(IQR): 27.2(26.1,29.6) vs. 27.3(26.1,29.1) vs. 27.2(25.9,28.8) (**D**) by smoking status (Median(IQR): 27.1(25.8,28.8) vs. 27.4(26.2,29.6) (**E**) by alcohol drinking (Median(IQR): 27.1(25.8,28.8) vs. 27.5(26.4,29.5) (**F**) oral hygiene (Median(IQR): 27.6(26.2,29.7) vs. 27.0(25.8,28.7)

The distribution of LI was analyzed according to disease status, age, BMI, smoking status, alcohol drinking, and oral hygiene. As shown in Fig. 2, LI was higher in the patient group (M = 27.6, IQR: 27.3–27.9) than in the control group (M = 28.2, IQR: 27.9–28.5). The results showed that younger subjects had a higher LI than older subjects. The distribution of LI was also significantly different across different smoking, alcohol drinking, and oral hygiene statuses.

Next, logistic regression was performed to assess the association between BMI, LI and oral cancer. As shown in Table 3, an inverse association between BMI and oral cancer risk was observed when BMI was treated as a continuous variable in Model 1, Model 2, and Model 3 (*OR* = 0.84, 95% *CI*: 0.80–0.88; *OR* = 0.84, 95% *CI*: 0.79–0.89; *OR* = 0.85, 95% *CI*: 0.80–0.89). Compared with normal BMI (18.5 ~ 23.9), the *ORs* of BMI < 18.5 kg/m^2^ and BMI ≥24 kg/m^2^ were 2.37 (95% *CI*: 1.25–4.50) and 0.54 (95% *CI*: 0.39–0.73), respectively, in Model 1. After adjustment for age, sex, residence, marital status, occupational activity and family history of cancer in Model 2, the association of BMI with oral cancer risk was more significant for BMI < 18.5 kg/m^2^ (*OR* = 2.45, 95% *CI*: 1.26–4.76) and BMI ≥24 kg/m^2^ (*OR* = 0.56, 95% *CI*: 0.41–0.78). In Model 3, where smoking, alcohol consumption, oral hygiene, LI, and diabetes were further adjusted, the effects of BMI < 18.5 kg/m^2^ (*OR* = 2.47, 95% *CI*: 1.25–4.89) and BMI > 24 kg/m^2^ (*OR* = 0.59, 95% *CI*: 0.43–0.83) on oral cancer risk were still significant. The association between LI and oral cancer was also examined using logistic regression. In the crude model, a higher risk of oral cancer was observed in the top tertile (*OR* = 2.27, 95% *CI*:1.59–3.24, *P*_trend_ < 0.001) than in the bottom tertile. This association was slightly attenuated in Model 2 (*OR* = 2.08, 95% *CI*: 1.45–2.99, *P*_trend_ < 0.001). The association was also slightly attenuated, but the association was still statistically significant after further adjusting for smoking, drinking, oral hygiene, BMI, and diabetes (*OR* = 1.99, 95% *CI*: 1.36–2.94, *P*_trend_ < 0.001). A positive and significant association was observed when LI was treated as a continuous variable in the crude model, Model 1 (*OR* = 1.07, 95% *CI*: 1.02–1.12; *OR* = 1.05, 95% *CI*: 1.01–1.11).

**Table 3:** Association between BMI, lipophilic index and risk of oral cancer

	n (control/case)	Model 1 *OR* (95%*CI*)	Model 2 *OR* (95%*CI*)	Model 3 *OR* (95%*CI*)
BMI				
< 18.5	14/39	2.37 (1.25,4.50)	2.45 (1.26,4.76)	2.47 (1.25,4.89)
18.5 ~ 23.9	195/229	1.00(ref.)	1.00(ref.)	1.00(ref.)
> 24	178/112	0.54 (0.39,0.73)	0.56 (0.41,0.78)	0.59 (0.43,0.83)
*P*_*trend*_		< 0.001	0.001	0.004
BMI^a^		0.84 (0.80,0.88)	0.84 (0.79,0.89)	0.85 (0.80,0.89)
Lipophilic index				
Tertile 1	148/120	1.00(ref.)	1.00(ref.)	1.00(ref.)
Tertile 2	149/119	1.08 (0.76,1.54)	1.02 (0.72,1.46)	0.81 (0.66,1.39)
Tertile 3	90/141	2.27 (1.59,3.24)	2.08 (1.45,2.99)	1.99 (1.36,2.94)
*P*_*trend*_		< 0.001	< 0.001	< 0.001
Lipophilic index^a^	27.59/28.17	1.07 (1.02,1.12)	1.05 (1.01,1.11)	1.05 (0.99,1.10)

^a^BMI or lipophilic index score treated as a continuous variable

Mode l was a crude model

Model 2 was adjusted for age, sex, education, residence, marital status, occupational activity and family history of cancer

Model 3 was further adjusted for smoking status, alcohol drinking, oral hygiene, and history of diabetes. Lipophilic index and BMI were further adjusted in anther model

**Fig. 3:**
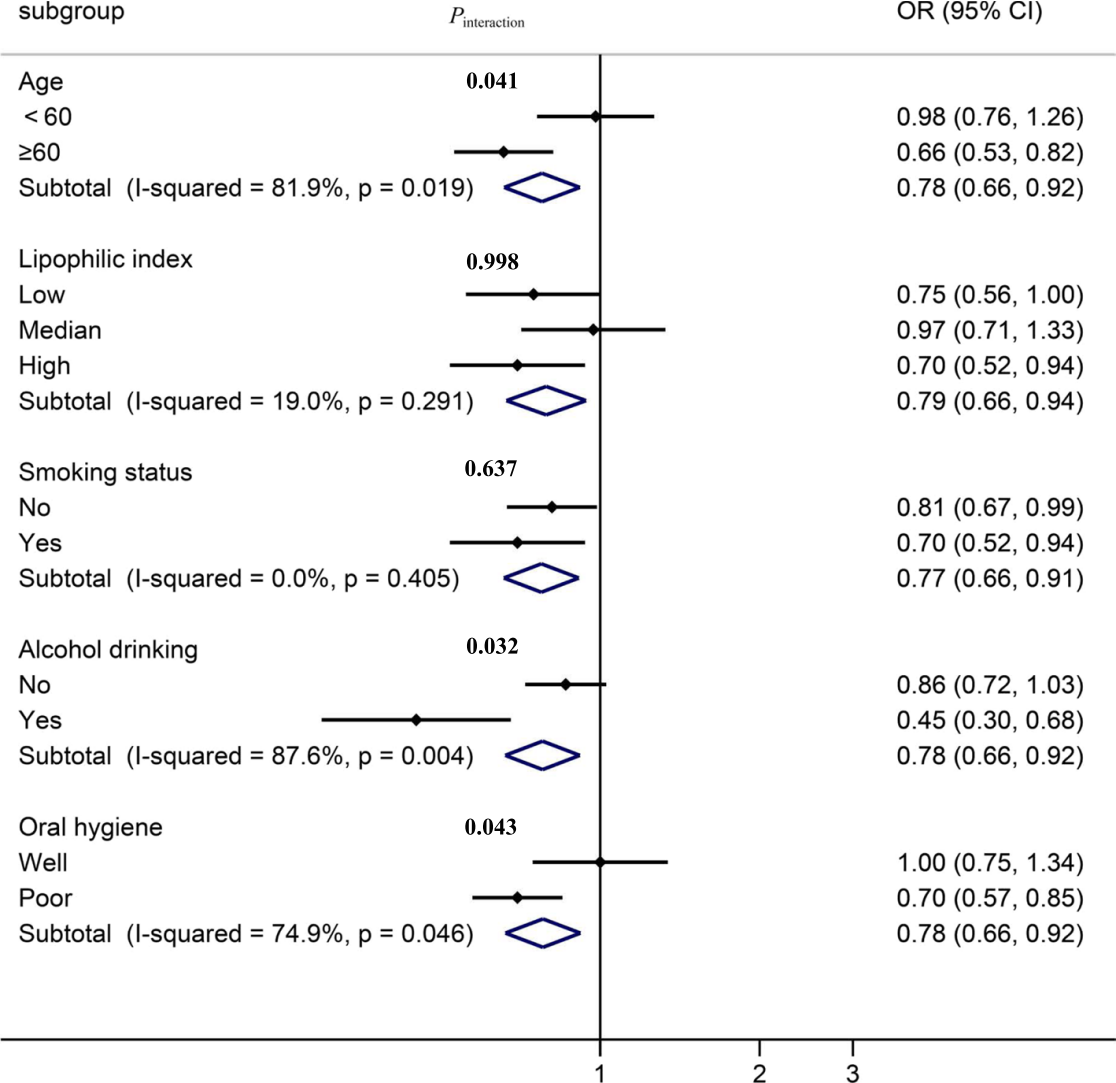
Association between the BMI and oral cancer by stratified analysis

The association between BMI and oral cavity cancer by stratified analysis was shown in Fig. 3. An inverse and significant association was observed between BMI and oral cancer when stratified by smoking status, and a strong effect was observed in smokers (*OR*_*tertile3v.1*_ = 0.70, 95% *CI*: 0.52–0.94). Associations of BMI and oral cancer remained significant among subjects ≥60 years (*OR*_*tertile3v.1*_ = 0.66, 95% *CI*: 0.53–0.82) and among subjects with poor oral hygiene (*OR*_*tertile3v.1*_ = 0.70, 95% *CI*: 0.57–0.85) compared with subjects aged < 60 years and subjects with better oral hygiene. When stratified by LI, an inverse and significant association was only observed in subjects with high LI (*OR*_*tertile3v.1*_ = 0.70, 95% *CI*: 0.52–0.94), and no interaction was shown between BMI and LI (*P*_interaction_ = 0.998). Moreover, the results of the stratified analysis between LI and oral cancer were shown in Supplementary Fig. 1. There was a positive association between LI and oral cancer when stratified by BMI and alcohol drinking and a strong effect between LI and oral cancer risk was observed among overweight or obese participants (*OR*_*tertile3v.1*_ = 1.46, 95% *CI*: 1.05–2.03). In addition, a stronger association was observed in non-smokers (*OR*_*tertile3v.1*_ = 1.52, 95%*CI*: 1.19–1.94) than in subjects who smoked.

The results of selecting covariates in mediation analysis were shown in Supplement Table 1. The multivariable analysis suggested that BMI, sex, age, and occupational activity were associated with LI, and the outcome model for oral cancer indicated that education, alcohol drinking, family history of cancer, and diabetes were significant. These significant covariates in the two models were considered simultaneously in the mediation analysis. Finally, the mediation effect of LI on the association between BMI and oral cancer risk was shown in Table 4. The results showed that LI could mediate the association between BMI and oral cancer with a significant percentage of 7%. The direct effects (BMI → oral cancer) and indirect effects (BMI → LI → Oral cancer) were presented as *ORs* of 0.884 (95% *CI*: 0.801, 0.889) and 0.986 (95% *CI*: 0.973,0.998), respectively.

**Table 4:** The effects of the BMI on oral cancer risk through lipophilic index

Effect^a^	β	SE	95%CI	
			LL	UL
Indirect (BMI → lipophilic index→oral cancer	−0.014	0.007	−0.027	− 0.001
*OR*s for indirect effect	0.986 (0.973,0.998)			
Percent of indirect effect	7%			
Direct (BMI → oral cancer)	−0.169		−0.222	−0.117
*OR*s for the direct effect	0.844 (0.801,0.889)			
Percent of direct effect	93%			

^a^Covariate includings: sex, age, occupational activity, education, alcohol drinking, family history of tumor, diabetes

## Discussion

The results of the current case–control study indicated that the oral cancer group was more likely to have a low proportion of overweight or obese patients than the control group, and the distribution of the FA profile in erythrocyte membranes differed between oral cancer patients and control participants. Erythrocyte membrane LI, which was derived from both FA melting points and FA composition, was associated with the risk of oral cancer. Moreover, erythrocyte membrane LI may mediate the association between BMI and oral cancer risk.

In studies conducted in Europe and North America, a lower BMI was observed to be linked to a higher risk of head and neck cancer (HNC) than a normal BMI [25–27]. Moreover, being overweight or obese was linked to a lower risk of HNC than being of normal weight [28]. In a pooled analysis by the International HNC Cancer Epidemiology Consortium [25], which included 17 case–control studies, low BMI was associated with a higher risk of HNC, while overweight or obesity was associated with a lower risk. In China, a large-scale study of 921 cases and 806 controls in eight centers investigated the role of BMI on HNC risk. The results showed that overweight and obesity were associated with a lower risk of HNC, while lean individuals had a higher risk of HNC [8]. Similar to previous studies, the current results also showed that BMI was inversely associated with oral cancer risk, and overweight or obesity was associated with a decreased risk of oral cancer.

The mechanism of the effect of BMI on oral cancer remains largely unknown, and one possible explanation is the dysregulation of lipid metabolism. In weight loss individuals, FA metabolism is influenced by weight loss through enzyme activity such as stearoyl-CoA desaturase (SCD) [29]. Obesity is characterized by metabolic disorder which is linked to perturbation of FA balance [30]. It has been reported that obesity tends to be a proinflammatory condition with changes in the FA composition of erythrocyte membranes [31]. A study of normal, overweight, and obese participants found that the FA profile of the erythrocyte membrane was different between the normal weight and obese groups [32]. Obesity associated with lipid accumulation was a major risk for insulin resistance [33], which was closely related to membrane fluidity [34] and FA profile [35].

Alterations in the FA composition of erythrocyte membranes were linked to an increased risk of cancer development [36, 37]. In a prospective study conducted in China, the FA profile of erythrocyte membranes in gastrointestinal cancer patients was found to be significantly different from that in healthy controls [38]. A case–control study within the EPIC Cohort Study discovered that a specific erythrocyte membrane phospholipid FA profile, possibly reflecting both a complicated dietary pattern and altered FA metabolism, was related to progressed colorectal adenoma risk [39]. Mouillot et al. found differences within the FA profile of the erythrocyte membranes of cirrhotic patients with hepatocellular carcinoma and cirrhotic controls without hepatocellular carcinoma [17]. An association between the erythrocyte membrane FA profile and cancer risk has also been found in other cohort studies [40–42]. The erythrocyte membrane FA profile differed in the presence of oral cancer. n-3 PUFAs were significantly decreased while oleic acid (C18:1 n-9) were significantly increased in oral cancer patients [43]. In the current study, different distributions of FA profiles in erythrocyte membranes were also observed between oral cancer patients and control participants.

However, the health effects of individual FAs even from the same category were heterogeneous, since the current FA classification was mainly based on structural properties instead of biological properties, such as the affinity between FA molecules and their fluidity. Therefore, a new LI was introduced in the current study and a positive association between LI and oral cancer risk was found. The LI was an overall estimate of the biological properties (melting point) and quantity of FA. LI was created to summarize the concentrations of individual FAs in a record that might be utilized to estimate the profile of FAs in biological samples such as erythrocytes. FA composition could influence skeletal cell membrane function with the aid of altered membrane fluidity, ion permeability, and insulin receptor binding and affinity [44]. A higher LI usually indicates poor membrane permeability and fluidity [13] and may affect the membrane’s physiological function [45]. Fluidity is a critical mediator that links the metabolism and consumption of FAs with cancer risk, and is related to several cellular functions including the properties of certain membrane-bound enzymes, mediator transport, endocytosis, phagocytosis, depolarization-dependent exocytosis, immunology and chemotherapeutic cytotoxicity, prostaglandin production and cell growth [46]. Altered insulin sensitivity and lipotoxicity are mechanisms that may link membrane fluidity to oral cancer risk [22]. Insulin resistance can stimulate cell proliferation and inhibit apoptosis, thus contributing to tumor promotion and metastasis through signal transduction of the insulin-like growth factor-1 receptor pathway [47–49]. In addition, the potential influence of lipids on neoplastic development may also be due to their influence on the metabolism of neoplastic cells, the function of lipids as intercellular messengers or as mediators of the inflammatory reaction [50]. Consistent with a previous study, a significant association between erythrocyte membrane FA profile-derived LI and oral cancer risk was observed in our study population.

Given that the FA profile was correlated with both BMI and oral cancer, it is plausible that FA composition could be a causal link between BMI and oral cancer, which was confirmed in the current study with erythrocyte membranes mediating 7% of the association between BMI and oral cancer. Previous studies [51, 52] have reported that obesity affects insulin resistance and consequently is involved in increasing the circulating level of free FA, which affects the tumor immune microenvironment through lipotoxicity. Balaban et al. [53] elucidated the role of FA metabolism in obesity and cancer and indicated that obesity affects tumorigenesis by interfering with the microenvironment of FA metabolism. For example, obesity altered the expression of CD36, which was correlated with FA transport [54] and has been associated with cancer progression. FA-binding proteins have been associated with FA intracellular transport, and obesity may increase the expression of FA-binding proteins and thus play an important role in cancer progression.

### Comparisons with other studies

Previous studies have revealed the association between BMI [25–27] and cancer as well as the association between changes in the FA profile in the cell membrane and cancer [36, 37]. In addition to findings reported by a previous study, the potential mediating effects of the membrane FA profile on the association of BMI with carcinogenesis were explored. In addition, the LI instead of individual FA levels was used in the current study. LI, which is calculated using both the melting point and level of individual FAs, is a better indicator of membrane functions.

### Strength and limitations

The strengths of this study include the following: 1) A comprehensive measurement of the FA profile of erythrocyte membranes was used to explore the relationship between membrane FA composition and oral cancer instead of using individual FA species. 2) Mediation analysis was performed to reveal the potential role of membrane FA on the association of BMI and oral cancer.

There were several limitations of our study. The melting points of several FAs were not available in the LipidBank database and hence could not be considered in the calculation of the LI. However, the levels of these FAs in the membrane were very low at less than 0.5%. Furthermore, this was a hospital-based, single-centre study with a case–control design. Large-scale prospective studies are needed to corroborate our findings.

## Conclusion

In summary, the distribution of the FA profile in erythrocyte membranes differed between the oral cancer patients and the control group. LI, which captures the overall lipophilicity of major FAs of erythrocytes, was positively associated with the risk of oral cancer. Associations between BMI and oral cancer risk can be explained, at least in part, by LI. This study revealed the potential role of membrane FA profile in oral cancer and may provide new perspectives in the development of new biomarkers and therapeutic targets for oral cancer.

## Supplementary Information

**Additional file 1: Supplement Figure 1.** Association between the lipophilic index and oral cancer by stratified analysis.

**Additional file 2: Supplement Table 1.** Multivariable generalized Linear Models of mediation of outcome models.

## Data Availability

The data that support the findings of this study are available from the corresponding author upon reasonable request.

## Abbreviations

LI: Lipophilic index
FA: Fatty acid
BMI: Body mass index
SFA: Saturated fatty acid
MUFA: Monounsaturated fatty acid
PUFA: Polyunsaturated fatty acid
OR: Odd ratio
HNC: Head and neck cancer
SCD: Stearoyl-CoA desaturase
