# Supplementary figures and images for: Mediation analysis of erythrocyte lipophilic index on the association between BMI and risk of oral cancer
Source: Lipids Health Dis. 2022 Oct 8;21:96. doi: 10.1186/s12944-022-01704-z (PMC9547469; doi:10.1186/s12944-022-01704-z)

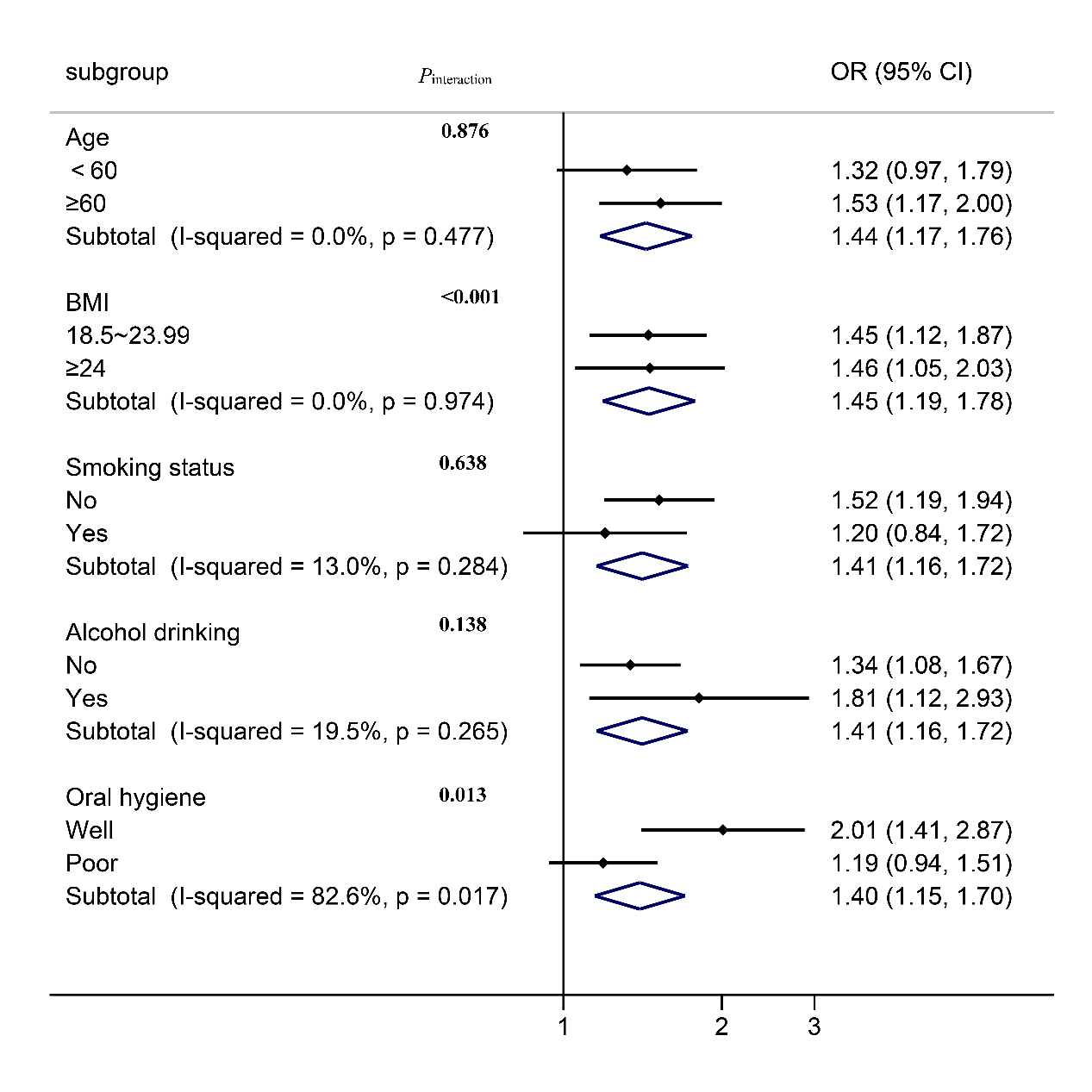

Supplement: Supplementary file 1 — Additional file 1: Supplement Figure 1. Association between the lipophilic index and oral cancer by stratified analysis. [file 12944_2022_1704_MOESM1_ESM.docx]
