# Supplementary material for: Mediation analysis of erythrocyte lipophilic index on the association between BMI and risk of oral cancer
Source: Lipids Health Dis. 2022 Oct 8;21:96. doi: 10.1186/s12944-022-01704-z (PMC9547469; doi:10.1186/s12944-022-01704-z)
Supplement: Supplementary file 2 — Additional file 2: Supplement Table 1. Multivariable generalized Linear Models of mediation of outcome models. [file 12944_2022_1704_MOESM2_ESM.docx]

**Supplement Table 1** Multivariable generalized Linear Models of mediation of outcome models

| Factors | β | SE | *P* | 95%CL |
| --- | --- | --- | --- | --- |
| Lipophilic index Model |  |  |  |  |
| BMI | -0.330 | 0.056 | 0.025 | (-0.439,-0.220) |
| Sex (Female vs Male) | 0.669 | 0.284 | 0.019 | (0.111,1.226) |
| Age (≥60 vs <60) | -1.105 | 0.225 | <0.001 | (-0.1547,-0.663) |
| Residence (Urban vs Rural) | 0.132 | 0.231 | 0.569 | (-0.322,0.585) |
| Education (High vs Low) | 0.080 | 0.254 | 0.100 | (-0.417,0.578) |
| Marital status (Others vs Married) | 0.211 | 0.385 | 0.583 | (-0.542,0.965) |
| Occupational activity |  |  |  |  |
| Moderate | 0.863 | 0.283 | 0.002 | (0.309,1.417) |
| Heavy | 0.512 | 0.306 | 0.094 | (-0.087,1.112) |
| Smoking status (Yes vs No) | 0.311 | 0.308 | 0.313 | (-0.294,0.916) |
| Alcohol drinking (Yes vs No) | -0.247 | 0.295 | 0.403 | (-0.826,0.332) |
| Family history of cancer (Yes vs No) | 0.255 | 0.295 | 0.386 | (-0.322,0.833) |
| Diabetes (Yes vs No) | -0.453 | 0.279 | 0.104 | (-1.000,0.093) |
| Oral hygiene (Poor vs Well) | -0.267 | 0.231 | 0.247 | (-0.720,0.185) |
| Oral cancer Model |  |  |  |  |
| Lipophilic index | 0.048 | 0.028 | 0.086 | (-0.007,0.102) |
| BMI | -0.163 | 0.082 | <0.001 | (-0.219,-0.108) |
| Sex (Female vs Male) | -0.412 | 0.219 | 0.107 | (-0.814,0.017) |
| Age (≥60 vs <60) | -0.282 | 0.175 | 0.107 | (-0.625,0.060) |
| Residence (Urban vs Rural) | -0.171 | 0.179 | 0.338 | (-0.522,0.179) |
| Education (High vs Low) | 1.224 | 0.204 | <0.001 | (0.825,1.623) |
| Marital status (Others vs Married) | 0.249 | 0.309 | 0.422 | (-0.359,0.856) |
| Occupational activity |  |  |  |  |
| Moderate | 0.219 | 0.217 | 0.313 | (-0.206,0.644) |
| Heavy | 0.013 | 0.235 | 0.955 | (-0.448,0.475) |
| Smoking status (Yes vs No) | 0.396 | 0.235 | 0.092 | (-0.064,0.857) |
| Alcohol drinking (Yes vs No) | 0.486 | 0.225 | 0.031 | (0.044,-.927) |
| Family history of cancer (Yes vs No) | 0.474 | 0.225 | 0.035 | (0.033,0.914) |
| Diabetes (Yes vs No) | -0.651 | 0.220 | 0.003 | (-1.082,0.219) |
| Oral hygiene (Poor vs Well) | -0.140 | 1.771 | 0.428 | (-0.488,0.207) |
